# Right Anterior Insula: Core Region of Hallucinations in Cognitive Neurodegenerative Diseases

**DOI:** 10.1371/journal.pone.0114774

**Published:** 2014-12-05

**Authors:** Frédéric Blanc, Vincent Noblet, Nathalie Philippi, Benjamin Cretin, Jack Foucher, Jean-Paul Armspach, François Rousseau, the Alzheimer's Disease Neuroimaging Initiative

**Affiliations:** 1 University Hospital of Strasbourg, Neuropsychology Unit, Neurology Service, Strasbourg, France; 2 University of Strasbourg and CNRS, ICube laboratory UMR 7357, FMTS (Fédération de Médecine Translationnelle de Strasbourg), Strasbourg, France; 3 University Hospital of Strasbourg, CMRR (Memory Resources and Research Centre), Strasbourg, France; 4 University Hospital of Strasbourg, Day Hospital of Geriatrics, Geriatrics Service, Strasbourg, France; University of California, San Francisco, United States of America

## Abstract

**Objectives:**

We investigated the neural basis of hallucinations Alzheimer's disease (AD) by applying voxel-based morphometry (VBM) to anatomical and functional data from the AD Neuroimaging Initiative.

**Methods:**

AD patients with hallucinations, based on the Neuropsychiatric Inventory (NPI-Q) (AD-hallu group; n = 39), were compared to AD patients without hallucinations matched for age, sex, educational level, handedness and MMSE (AD-c group; n = 39). Focal brain volume on MRI was analyzed and compared between the two groups according to the VBM method. We also performed voxel-level correlations between brain volume and hallucinations intensity. A similar paradigm was used for the PET analysis. “Core regions” (i.e. regions identified in both MRI and PET analyses, simply done by retaining the clusters obtained from the two analyses that are overlapping) were then determined.

**Results:**

Regions with relative atrophy in association with hallucinations were: anterior part of the right insula, left superior frontal gyrus and lingual gyri. Regions with relative hypometabolism in association with hallucinations were a large right ventral and dorsolateral prefrontal area. "Core region" in association with hallucinations was the right anterior part of the insula. Correlations between intensity of hallucinations and brain volume were found in the right anterior insula, precentral gyrus, superior temporal gyrus, and left precuneus. Correlations between intensity of hallucinations and brain hypometabolism were found in the left midcingulate gyrus. We checked the neuropathological status and we found that the 4 patients autopsied in the AD-hallu group had the mixed pathology AD and Dementia with Lewy bodies (DLB).

**Conclusion:**

Neural basis of hallucinations in cognitive neurodegenerative diseases (AD or AD and DLB) include a right predominant anterior-posterior network, and the anterior insula as the core region. This study is coherent with the top-down/bottom-up hypotheses on hallucinations but also hypotheses of the key involvement of the anterior insula in hallucinations in cognitive neurodegenerative diseases.

## Introduction

The reported prevalence of hallucinations in Alzheimer's disease (AD) patients varies from 0 to 25% depending on the study [1]. However, if one considers only studies that included neuropathologic diagnosis, the values range from 15 to 20% [2]. Hallucinations are consistently reported to be more frequent in Dementia with Lewy bodies (DLB) than in AD, with a prevalence of 75% in DLB [2], [3].

In previous studies of AD patients, hallucinations were related to posterior region changes, with a relative occipital atrophy [4], more occipital white matter hyperintensities [5], and enlarged ventricles [6], [7]. The brain structures responsible for such behavioral modifications have been more extensively explored in DLB, where hallucinations were found to be correlated to posterior decreased perfusion including left posterior cingulate and precuneus [8], but also angular gyri, right supramarginal gyrus and 4^th^ occipital gyrus [9]. Moreover, Taylor et al started hallucinations in DLB patients in one third of the cases using transcranial magnetic stimulation of the occipital lobe [10]. They found also that the severity of visual hallucinations were strongly correlated with occipital lobe excitability [10]. DLB patients with hallucinations were shown to have greater gray matter (GM) loss in the right inferior part of the frontal lobe [11]. PD patients with hallucinations compared to Parkinson's disease (PD) patients without, matched for cognitive status, exhibited grey matter atrophy in the cuneus, lingual and fusiform gyri, middle occipital lobe, inferior parietal lobule, and cingulate, paracentral and precentral gyri [12].

The mechanisms of visual hallucinations in cognitive neurodegenerative diseases such as AD, DLB, or PD are debated with numerous different hypotheses [13]. First the release phenomenon is a mechanism of disinhibition of neurons with spontaneous activity of them: particularly cholinergic denervation of occipital associative areas would induce hallucinations [14]. Against this hypothesis O'Brien et al have found an elevation of nicotinic receptor binding in such areas in DLB patients with hallucinations [15]. Visual disturbances or disturbances in visuo-cortical areas are also frequently supposed to be a core mechanism of hallucinations: many studies support this bottom-up visual processing hypotheses, with impaired visual processing and reduced activation in visual cortices [16], [17]. More recently, Diederich et al., suggested that visual hallucinations should be considered as a dysregulation of the gating and filtering of external perception and internal image production [18]. Collerton et al., developed the "Perception and Attention deficit Model": a combination of impaired attentional binding (top-down) and perceptual processes (bottom-up) [16]. Finally Shine et al., have proposed, but only for PD, the hypotheses that the difficulties to activate the dorsal attention network at the presentation of a visual stimulus, is responsible for a conflict resolution processed by neural networks unprepared to do it (default mode network and ventral attention network also named salience network), and thus hallucinations [19].

A better knowledge of the mechanisms of neuropsychiatric symptoms in AD, especially regarding hallucinations, is of importance since currently used antipsychotic treatments carry an increased long-term risk of mortality [20].

The aim of this study was to investigate the neural correlates of hallucinations in AD patients from the Alzheimer's Disease Neuroimaging Initiative (ADNI). We used a dual approach based (1) on the analysis of focal volume on AD brain MRI using voxel-based morphometry (VBM) to localize relative brain atrophy associated with the presence and intensity of hallucinations, and (2) on the analysis of functional data with fluorodesoxyglucose positron emission tomography (FDG-PET), using SPM to evaluate voxelwise brain metabolism modifications associated with the presence and intensity of hallucinations. We hypothesised that in AD patients with hallucinations, we would find more posterior (occipital) and anterior (frontal) atrophy and hypometabolism.

## Method

### Ethic

These data were analyzed anonymously. All subjects gave written, informed consent prior to participation through the local institutional review boards at participating institutions. For the purpose of this study we used the Alzheimer's Disease Neuroimaging Initiative (ADNI) data that were previously collected across 50 sites. Study subjects gave written informed consent at the time of enrolment for data collection and completed questionnaires approved by each participating site's Institutional Review Board (IRB). The complete list of ADNI sites' IRBs can be found in the link: http://adni.loni.ucla.edu/about/data-statistics/. Specifically, they are: Albany Medical College, Banner Alzheimer's Institute, Baylor College of Medicine, Boston University, Brigham and Women's Hospital, Butler Hospital Memory & Aging Program, Case Western Reserve University, Cleveland Clinic, Columbia University, Darthmouth – Hitchcock Medical Center, Dent Neurologic Institute, Duke University Medical Center, Emory University, Georgetown University, Howard University, Indiana University, Jefferson Hospital for Neuroscience, Johns Hopkins University, Mayo Clinic, Jacksonville, Mayo Clinic, Rochester, McGill University/Jewish General Hospital Memory Clinic, Medical University of South Carolina, Mount Sinai School of Medicine, Neurological Care of Central New York, New York University Medical Center, Northwestern University, Ohio State University, Olin Neuropsychiatry Research Center, Oregon Health and Science University, Parkwood Hospital, Premiere Research Institute, Rhode Island Hospital, Rush University Medical Center, Saint Joseph's Health Center, London, Ontario, Stanford University, Banner Sun Health Research Institute, Sunnybrook Health Sciences, University of Alabama, Birmingham, University of British Columbia, University of California, Davis, University of California, Irvine, University of California, Irvine-BIC, University of California, Los Angeles, University of California, San Diego, University of California, San Francisco, University of Kansas, University of Kentucky, University of Michigan, Ann Arbor, University of Nevada School of Medicine, Las Vegas, University of Pennsylvania, University of Pittsburgh, University of Rochester, University of Southern California, University of Texas Southwestern Medical Center, University of Wisconsin, Wake Forest University, Washington University St. Louis, Wein Center for Clinical Research and Yale University School of Medicine. The protocol was submitted to appropriate Boards and their written unconditional approval obtained and submitted to Regulatory Affairs at the Alzheimer's Disease Neuroimaging Initiative Coordinating Center (ADNI-CC) prior to commencement of the study.

### Subjects from the Alzheimer's Disease Neuroimaging Initiative Database

Data used in the preparation of this article were obtained from the Alzheimer's Disease Neuroimaging Initiative (ADNI) database (adni.loni.ucla.edu). The ADNI was launched in 2003 by the National Institute on Aging (NIA), the National Institute of Biomedical Imaging and Bioengineering (NIBIB), the Food and Drug Administration (FDA), private pharmaceutical companies and non-profit organizations, as a $60 million, 5-year public-private partnership. The primary goal of ADNI has been to test whether serial magnetic resonance imaging (MRI), positron emission tomography (PET), other biological markers, and clinical and neuropsychological assessment can be combined to measure the progression of mild cognitive impairment (MCI) and early Alzheimer's disease (AD). Determination of sensitive and specific markers of very early AD progression is intended to aid researchers and clinicians to develop new treatments and monitor their effectiveness, as well as lessen the time and cost of clinical trials.

The Principal Investigator of this initiative is Michael W. Weiner, MD, VA Medical Center and University of California – San Francisco. For up-to-date information, see www.adni-info.org.

ADNI is a large multicenter, longitudinal, observational trial taking place across the United States and Canada, launched in 2003, in which subjects with normal cognition, amnestic mild cognitive impairment (MCI), and mild AD are followed up with periodic neuropsychological testing, multiple imaging techniques, and fluid biomarkers. Subjects with complete baseline clinical datasets (n = 765; including 387 amnestic MCI, 200 controls and 178 mild AD) were included in the current study. They had a study partner able to provide an independent evaluation of the patient. They also had a modified Hachinski Ischemic Score ≤4 and a Geriatric Depression Scale (GDS, short form) <6. Subjects did not have other significant neurological disease, significant active psychiatric disorders, or alcohol or substance abuse within 2 years of screening. For full inclusion/exclusion criteria see http://www.adni-info.org.

AD subjects fulfilled these criteria: CDR score of 1.0 or 2.0 and met the National Institute of Neurologic and Communicative Disorders and Stroke and the Alzheimer's Disease and Related Disorders Association Work Group criteria (NINCDS/ADRDA criteria) for probable AD [21]. The patients fulfilled also the Dubois' research criteria for AD – the high specificity of which has been confirmed - [22], because all of them had memory impairment, atrophy of hippocampus on brain MRI, and abnormal CSF for all the subjects that underwent lumbar puncture (see table 1) [23].

**Table 1:** Clinical and Demographic Features of Alzheimer's Disease Patients with Hallucinations (AD-hallu), without Hallucinations (AD-c) and Healthy Elderly Controls (HC).

		AD-hallu, N = 39	AD-c, N = 39	HC, N = 39	Test statistic, P	Post *hoc* ^b^
Age, years^a^		76.0 (7.4)	76.4 (7.2)	78.8 (4.8)	F = 2.000, P = .140	
Years of education^a^		14.2 (3.5)	14.3 (2.9)	15.5 (2.6)	H = 4.757, P = .093	
Female/male		19/20	19/20	19/20	?^2^ = .069, P = .966	
MMSE score^a^		19.2 (5.0)	20.8 (4.1)	29.0 (0.9)	H = 77.747, P<.0001	HC>AD-hallu and AD-c
CDR		1.3 (0.5)	1.1 (0.3)	0.03 (0.2)	H = 91.940, P<.0001	HC<AD-hallu and AD-c
FAQ		20.6 (8.0)	16.6 (6.7)	0.15 (0.4)	H = 82.652, P<.0001	HC<AD-hallu and AD-c
Hallucinations (NPI-Q)		39	0	0	?^2^ = 117.000, P<.0001	
No. right-hander patients		33	35	37	?^2^ = 2.229, P = .328	
Parkinsonism		0	0	0		
No. (%) of subjects with CSF		24 (62%)	25 (64%)	39 (100%)		
Abeta1-42 (SD; No.<192 pg/ml)		137.9 (23.7; 24)	137.3 (37.6; 22)	250.2 (24.4;0)	F = 168.439, P<.0001	HC>AD-hallu and AD-c
P-Tau (SD; No.>23 pg/ml)		38.6 (19.4; 21)	50.0 (25.1; 23)	16.6 (3.2;0)	F = 32.393, P<.0001	HC>AD-hallu and AD-c
Tau (SD; No.>93 pg/ml)		115.7 (56.5; 13)	130.2 (60.1; 16)	52.0 (12.3;0)	F = 28.762, P<.0001	HC>AD-hallu and AD-c
No. of abnormal CSF biomarker (0/1/2/3)		0/3/8/13	1/3/5/16	39/0/0/0	H = 71.321, P<.0001	HC<AD-hallu and AD-c
No. of patients with brain MRI		39	39	39		
No. of patients with brain FDG-PET		19	20	21		
Features of patients with FDG-PET	Age^a^	75.8 (7.4)	76.3 (6.6)	78.9 (4.9)	F = 1.424, P = .249	
	Years of education^a^	14.3 (3.6)	14.7 (3.3)	14.9 (2.9)	H = 1.105, P = .576	
	Female/male	10/9	10/10	12/9	?^2^ = .215, P = .0898	
	MMSE score^a^	19.0 (5.5)	20.6 (4.5)	29.1 (.8)	H = 40.260, P<.0001	HC>AD-hallu and AD-c
	CDR	1.4 (.5)	1.2(.4)	.05 (.2)	H = 45.691, P<.0001	HC<AD-hallu and AD-c
	FAQ	20.4 (8.1)	17.3 (6.5)	.19 (.5)	H = 42.481, P<.0001	HC<AD-hallu and AD-c
	Hallucinations (NPI-Q)	19	0	0	?^2^ = 60.000, P<.0001	
	No. right-hander patients	16	19	20	?^2^ = 2.024, P = .363	
	Parkinsonism	0	0	0		
	No. (%) of subjects with CSF	11 (58%)	12 (60%)	21 (100%)		
	Abeta1-42 (SD; No.<192 pg/ml)	136.6 (26.3;11)	124.6 (27.8;12)	248.8 (25.8;0)	F = 110.560, P<.0001	HC>AD-hallu and AD-c
	P-Tau (SD; No.>23 pg/ml)	42.2 (20.7;11)	55.7 (26.5; 12)	16.7 (3.6;0)	F = 21.228, P<.0001	HC>AD-hallu and AD-c
	Tau (SD; No.>93 pg/ml)	121.0 (59.9; 6)	140.4 (56.4;9)	53.1 (12.7;0)	F = 19.101, P<.0001	HC>AD-hallu and AD-c
	No. of abnormal CSF biomarker (0/1/2/3)	0/0/5/6	0/0/3/9	21/0/0/0	H = 38.285, P<.0001	HC<AD-hallu and AD-c

a mean (standard deviation), ^b^Tukey post-hoc test for ANOVA (F), KruskallWallis post-hoc test on SPSS (H), there is no statistically significant difference between AD-hallu and AD-c. FDG-PET = fluorodesoxyglucose positron emission tomography; MMSE = Mini-Mental Status Examination; No. = number; NPI-Q = brief clinical form of the Neuropsychiatric Inventory.

In the ADNI cohort, evaluation of hallucinations was based on the brief clinical form of the Neuropsychiatric Inventory (NPI) [24]. The brief clinical form of the NPI, also called NPI-Q, is a retrospective (previous month) study-partner-administered questionnaire covering 12 neuropsychiatric symptom domains: delusions, hallucinations, agitation/aggression, dysphoria/depression, anxiety, euphoria/elation, apathy/indifference, disinhibition, irritability/lability, aberrant motor behaviors, nighttime behavioral disturbances, and appetite/eating disturbances. The scripted NPI questionnaire includes a written screening question for each domain. For hallucinations, the wording of the screening question is: “Does he/she have hallucinations, or false visions, or voices? Does he/she seem to hear or see things that are not present?” (see https://www.alz.washington.edu/npiq/). Neuropsychiatric manifestations within a domain are rated by the study partner in terms of severity/intensity on a three point scale: 1: mild (noticeable but not significant change), 2: moderate (significant but not a dramatic change), 3: severe (very marked or prominent; a dramatic change).

Among the 565 patients, AD patients with hallucinations (AD-hallu group) were compared to AD patients without hallucinations (AD-c group) matched for age, sex, educational level, handedness and MMSE (Table 1). Forty-nine AD patients were found to have hallucinations according to the NPI-Q. However, only 39 patients were included because the 6 other patients did not have brain MRI and/or FDG-PET at the same time as the NPI-Q, and 4 patients were diagnosed as AD-MCI. The comparison of AD-hallu and AD-c for the neuropsychiatric symptom domains of the NPI-Q logically showed a clear difference for the item ‘hallucinations’ (χ^2^ = 78.000, P<.0001), a difference for the domains ‘agitation/aggression’ (χ^2^ = 4.255, P = .039), ‘anxiety’(χ^2^ = 7.429, P = .006), and ‘aberrant motor behaviors’ (χ^2^ = 5.769, P = .016), but no difference for the other domains. There were no significant differences between the AD-hallu group and the AD-c group in terms of psychotropic treatment (antidepressant, benzodiazepine and antipsychotic drugs), or AD treatment (cholinesterase inhibitors and memantine). Finally 39 healthy elderly controls matched for age, gender, educational level and handedness were also considered.

Brain FDG-PET and MRI images were downloaded from the ADNI LONI website in NIFTI format.

### MRI Acquisition

Standard 1.5T T1-weighted images obtained using volumetric 3D MPRAGE protocol with resolutions ranging from 0.9 mm×0.9 mm×1.20 mm to 1.3 mm×1.3 mm×1.20 mm were included from the ADNI database. For detailed information on the MRI protocols and preprocessing steps, see Jack et al [25]. It is of importance to notice that a major effort was devoted in establishing the MRI protocol specifications to standardize 3D T1-weighted sequences across sites and platforms in order to enable the scientific community to carry out relevant multicentric morphometric study (see [25] for details). In our work, we choose to restrict our analysis to 1.5 MRI scans only (3T MRI examination were discarded) in order to have even more consistent data.

### FDG-PET Acquisition

Subjects were scanned after a 4-hour fast (water only). Plasma glucose had to be ≤180 mg/dl for FDG to be injected. An intravenous catheter was placed in one arm for injection of 18F-FDG. Imaging began at 30 minutes post-injection, and the scan was acquired as six 5-minute frames. For detailed information on the MRI protocols and preprocessing steps, see Langbaum et al., [26].

### Image Analysis

#### MRI image analysis

For MRI analysis, 39 patients with hallucinations (AD-hallu group) were compared to 39 control patients (AD-c group). Each of these two groups was preliminary compared to the group of 39 healthy elderly controls (HC). Focal brain atrophy of gray mater (GM) and white matter (WM) was compared between groups using the VBM (voxel-based morphometry) method provided in SPM12b (Statistical Parametric Mapping, Wellcome Department of Cognitive Neurology, London, U.K.) and ran under Matlab R2010a software (The MathWorks, Natick, MA). We also performed voxel-level correlations between GM/WM atrophy and hallucinations intensity.

Voxel-based morphometry analyses included image pre-processing and statistical analyses. Anatomical MRI images were spatially pre-processed using standard procedures [27]. All T1 structural images were first segmented, bias corrected and spatially normalized to the Montreal Neurological Institute (MNI) space using an extension of the unified segmentation procedure [28] that includes six classes of tissue. Spatial normalization is then refined using DARTEL algorithm [29] and a customized brain template is built from the set of all images.

The VBM analysis was done on modulated gray matter and white matter images (i.e., GM and WM segmentation maps were multiplied by the Jacobian of the transformation in order to account for the variation of volume induced by the transformation). These images were smoothed with a Gaussian kernel (FWHM: 8 mm).

Then, we performed voxel-level comparison of GM (resp. WM) atrophy on the one hand between each of the AD groups (AD-hallu and AD-c) and the HC-group and on the other hand between the AD-hallu group and the AD-c group using a two-sample t-test while considering age and total amount of GM (resp. WM) as covariates. Statistical maps were thresholded either with P_FWE_<0.05 corrected for familywise error (for the comparisons between AD groups and HC-group) or *P*<0.001 without p-value correction (for the comparison between AD-hallu and AD-c) and with a minimum cluster size of 25 voxels. Additionally, we performed voxel-level correlations between GM (resp. WM) atrophy and the intensity of hallucinations (NPI-Q) for the AD-hallu group using the intensity of hallucinations as covariate of interest and age and total amount of GM (resp. WM) as confounders. Corresponding statistical maps were also thresholded with *P*<0.001 (uncorrected p-value) and with a minimum cluster size of 25 voxels (200 mm^3^).

#### PET image analysis

For FDG-PET analysis, 19 patients with hallucinations (AD-hallu subgroup) were compared to 20 control patients (AD-c subgroup). Each group was also compared to 20 healthy elderly controls (HC subgroup)

Focal brain metabolism was compared between groups and voxel-level correlations between brain metabolism and hallucinations intensity were performed within AD-hallu group. To this end, all PET images were spatially normalized to the Montreal Neurological Institute (MNI) space. This was done by first registering each PET image on the corresponding MRI image for each subject using an affine transformation. Then, the transformations estimated from MRI analysis using Dartel algorithm were then applied on the affinely registered PET images. By this way, we benefit from the structural information of MRI data to accurately match all PET images. These normalized images were then smoothed with a Gaussian kernel (FWHM: 8 mm).

We performed voxel-level comparisons of brain metabolism between either the AD-hallu group or the AD-c group with the HC group, and between the AD-hallu group and the AD-c group using a two-sample t-test while considering age and mean brain metabolism as nuisance variables. Statistical maps were thresholded with *P*<0.001 (uncorrected p-value) and with a minimum cluster size of 25 voxels. Additionally, we performed voxel-level correlations between focal brain metabolism and the intensity of hallucinations (NPI-Q) for the AD-hallu group using a one-sample t-test with the intensity of hallucinations as the covariate of interest and age and mean brain metabolism as confounders. Corresponding statistical maps were also thresholded with *P*<0.001 (uncorrected p-value) and with a minimum cluster size of 25 voxels.

#### MRI and PET image analysis: “core regions”

We define as "core regions" the regions that were identified in both MRI and PET analyses. This is simply done by retaining the clusters obtained from the two analyses that are overlapping. We looked for these "core regions" either for the comparison between AD-hallu and AD-cor for the correlation analyses with the intensity of hallucinations.

MNI Space Utility (MSU, http://www.ihb.spb.ru/~pet_lab/MSU/MSUMain.html), xjView software (http://www.alivelearn.net/xjview8/) and Talairach software (http://www.talairach.org/index.html ) were used to create reports about cluster localization in terms of Talairach Daemon anatomical region labels for both MRI and PET analysis.

### Neuropathological status

Based on the article of Toledo et al., [30] we checked the neuropathological status of the patients included in this article to better understand the origin of hallucinations in patients.

### Statistical analysis

The Statistical Package for Social Sciences software (SPSS ver. 21.0.0.0, http://www-01.ibm.com/software/analytics/spss/) was used for further statistical evaluation as required. Where appropriate, differences in demographic and clinical data were assessed using parametric (ANOVA, t-tests) and non-parametric tests (Kruskall-Wallis H, Mann-Whitney U). Post-*hoc* analysis were applied: Tukey post-*hoc* test for ANOVA and Kruskall-Wallis post-*hoc* test for H. For categorical measures, χ^2^ tests were applied. For each test statistic, a probability value of P<0.05 was regarded as significant.

## Results

### Comparison of AD patients and healthy controls

**Figure 1:**
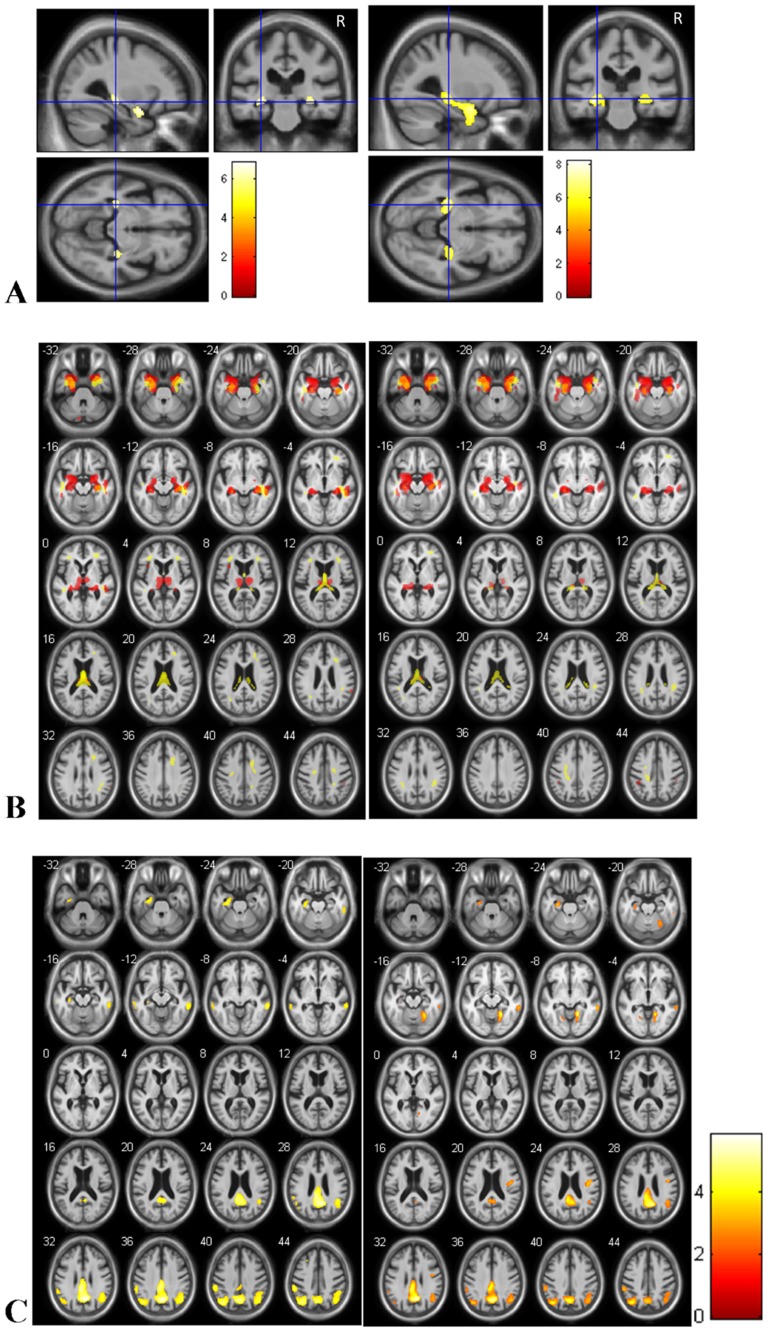
Brain regions of relative atrophy of AD patients with hallucinations compared to healthy controls (A and B left) and AD patients without hallucinations compared to healthy controls (A and B right). Brain regions of relative hypometabolism of AD patients compared to healthy controls (C). (A: for gray matter only: T = p<0.05, correction FWE, minimum cluster size = 25 voxels; B: T = p<0.001, without correction, minimum cluster size = 25 voxels; C. T = p<0.001, without correction, minimum cluster size = 25 voxels) A. The relative atrophy involves hippocampi and amygdalas for the two AD groups, for the analysis with correction. B. The relative atrophy of gray matter (red) and white matter (yellow) involves also input and output pathways of the hippocampi including amygdalas, entorhinal cortex, fornix, thalami and cingulate gyrus. C: Relative hypometabolism (FDG PET) of AD-hallu (left) and AD-c (right) compared to healthy controls. Right is right.

Comparison with FWE correction (P<0.05) of GM of AD-hallu patients with healthy controls showed significant relative atrophy in hippocampi and amygdalas (Figure 1A left). The same regions were found for the comparison of AD-c patients and healthy controls (Figure 1A right). The whole cluster size of voxels is of 5554 when comparing AD-c to healthy controls, and of 1389 when comparing AD-hallu to healthy controls.

Comparison with FWE correction (P<0.05) of WM of AD-hallu patients with healthy controls showed significant relative atrophy in WM at the vicinity of the anterior part of left hippocampus and amygdalae and fornix (data not shown). The same regions were found for the comparison of AD-c patients and healthy controls but bilaterally.

Without P-value correction (P<0.001), the comparison of GM and WM of AD-hallu patients with healthy controls showed significant relative atrophy in the whole hippocampi and input and output pathways of the hippocampi including amygdalas, entorhinal cortices, fornix, thalami and cingulate gyri (Figure 1B left). The same regions were found for the comparison of AD-c patients and healthy controls (Figure 1B right).

Comparison with FWE correction (p<0.05) of FDG-PET of AD-hallu patients with healthy controls did not show any relative hypotemabolism, nor hypermetabolism. Comparison with FWE correction (p<0.05) of FDG-PET of AD-c patients with healthy controls showed little hypometabolism of the right precuneus (number of voxels 54, T = 5.80, x = 9, y = −60, z = 31.5). Comparison without correction (p<0.001) showed hypometabolism of the temporal, parietal and frontal lobes (figure 1C).

### AD brain focal atrophy and hallucinations

**Figure 2:**
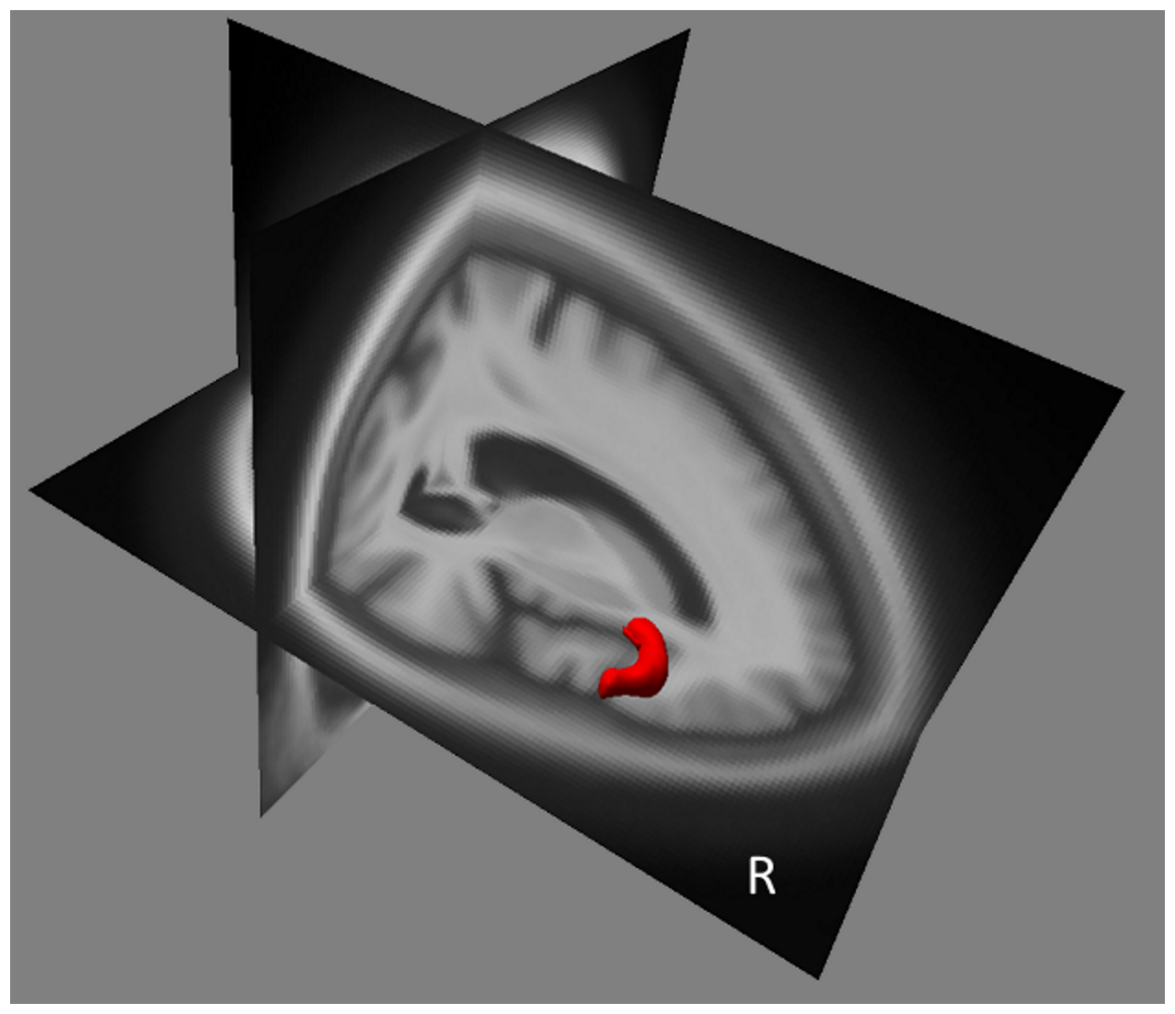
Brain Regions of Relative Grey Matter Atrophy Associated with the Existence of Hallucinations in AD Patients. Relative atrophy of GM (red): right anterior part of insula and inferior frontal gyrus. (T = p<0.001, uncorrected, minimum cluster size = 25 voxels).

The main brain regions that showed significant relative atrophy in association with hallucinations in AD patients (AD-hallu minus AD-c) were in WM the lingual gyri of the occipital lobe (right more than left), (Table 2), and in GM: the anterior part of the right insular cortex and a discrete part of the left superior frontal gyrus (Table 2 and Figure 2).

**Table 2:** A. Brain Regions that Show Significant Relative Atrophy in Relation to the Presence of Hallucinations in AD patients.

						Coordinates (mm, MNI)			
	Brain regions	BA vicinity	Side	No. Voxels	T value	x	y	z	p
**A**	Insula, anterior part and inferior frontal gyrus (GM)	13/45/47	R	594	4.13	45	28	6	0.000047
	Frontal lobe, superior frontal gyrus (GM)	6	L	70	3.72	−9	27	61	0.00019
	Occipital lobe, lingual gyrus (WM)	18	R/L	113	3.62	7.5	−86	−4	0.00026
**B**	Frontal lobe, precentral gyrus (GM)	6	R	86	4.52	37	−8	32	0.0000034
	Insula, anterior part (GM)	13	R	75	3.77	34	10	14	0.00003
	Temporal lobe, superior temporal gyrus (GM)	21	R	69	4.02	51	−6	−13	0.00010
	Parietal lobe, precuneus (WM)	7	L	63	4.37	−14	−57	71	0.0000053

T = p<0.001, uncorrected, minimum cluster size = 25 voxels; BA = Brodmann's area; L = left; GM = gray matter; No. = number; R = right; sup.  = superior; WM = white matter.

AD patients with hallucinations have been compared to matched (age, sex, handedness, educational level and MMSE) AD patients without hallucinations. **B. Correlation between Hallucination Intensity (NPI-Q) and Regional Brain Volume (MRI).** Underlined brain regions are “core regions” of hallucinations in AD (see Methods).

### AD brain focal atrophy and intensity of hallucinations

One correlation was found between hallucination intensity (NPI-Q, score 1 to 3) and regional WM volume in the AD-hallu group: the left precuneus (Table 2B).

Correlations were found between hallucination level and regional GM volume in some brain regions: Right anterior part of the insula, right precentral gyrus, and right superior temporal gyrus (Table 2B).

### AD brain metabolism and hallucinations

**Figure 3:**
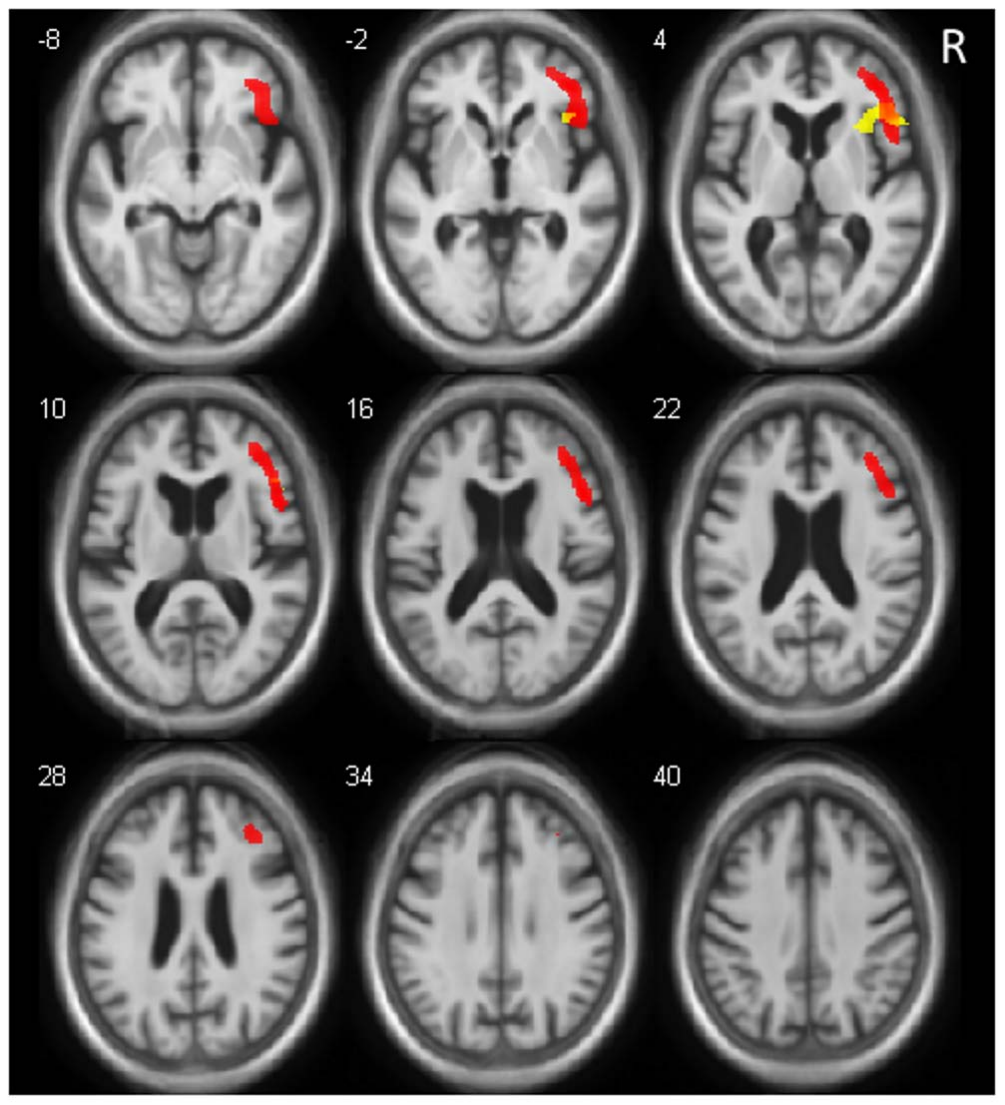
Brain Regions with Relative Hypometabolism (red) and Relative Gray Matter Atrophy (yellow) in Relation with Hallucinations. Relative hypometabolism of right inferior frontal gyrus, middle frontal gyrus (including orbital part), and insula (anterior part) (slices z = −8 to 28). Core regions correspond to the common part of relative hypometabolism and atrophy, including right inferior frontal gyrus and a part of right anterior insula. (For MRI and FDG PET, p<0.001, uncorrected, minimum cluster size = 25 voxels). Right is right.

The comparison of brain metabolism between the two groups showed that hallucinations were associated with extensive hypometabolism in the right frontal lobe (anterior part including the orbito-frontal region and insula; Figure 3 and Table 3). This comparison also highlighted patchy hypermetabolism in the left hemisphere including superior frontal gyrus, fusiform gyrus, post-central gyrus, and the supramarginal gyrus and precuneus in the AD-hallu group (Table 3).

**Table 3:** Brain Regions of Relative Hypometabolism Associated with the Existence of Hallucinations (Hypo), and Brain Regions of Relative Hypermetabolism Associated with the Existence of Hallucinations (Hyper).

							Coordinates MNI (mm)		
	Brain Regions	BA Vicinity	Side	No. Voxels	T value	x	y	z	p
**Hypo**	Frontal lobe, inferior, middle frontal gyrus (orbital), and insula (ant)	45/47/44/10/9	R	3625	4.56	46	25	2	0.000031
**Hyper**	Frontal lobe, superior frontal gyrus	6	L	347	4.52	−24	−11	69	0.000036
	Temporal lobe, fusiform gyrus	37	L	91	3.92	27	−41	−20	0.00020
	Parietal lobe, postcentral gyrus and precuneus	7	L L	90	3.79	−15	−53	74	0.00029
	Parietal lobe, postcentral and supramarginal gyri	40		353	3.71	−51	−21	33	0.00037

T = p<0.001, uncorrected, minimum cluster size = 25 voxels; ant = anterior; BA = Brodmann's area; L = left; R = right; NA = not applicable; No. = number.

Underlined brain regions are “core regions” of hallucinations in AD (see Methods).

### AD brain metabolism and intensity of hallucinations

**Figure 4:**
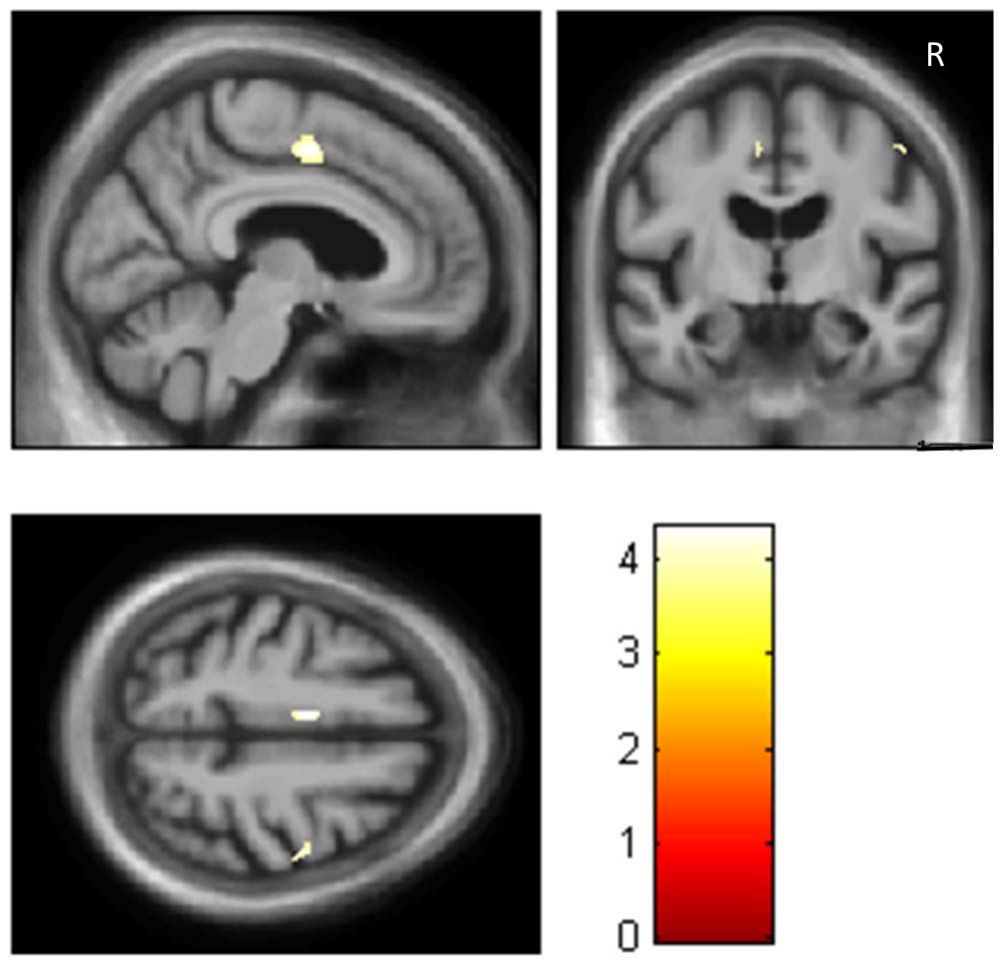
Alzheimer's Disease Brain Hypometabolism and Intensity of Hallucinations. Correlations between brain hypometabolism and intensity of hallucinations in the left midcingulate gyrus (BA 24) (and discretely right precentral gyrus, BA6). (T = p<0.001, uncorrected, minimum cluster size = 25 voxels). Right is right.

Correlations between brain metabolism and intensity of hallucinations revealed a hypometabolism in the left limbic lobe, cingulate gyrus (T = 4.34, number of voxels = 100; BA 24 figure 4), and in the right pre -central gyrus (T = 4.160, number of voxels = 31).

### Core regions associated with hallucinations in AD

The common overlapping regions of focal changes in AD brain volume and metabolism and hallucinations were the right anterior insula and inferior frontal gyrus (Table 2 and figure 3).

We did not find any overlapping regions in which changes in AD brain volume and metabolism were correlated with intensity of hallucinations.

### Neuropathological results

Among the 39 AD-hallu patients, 4 were autopsied (patients 565, 691, 723, 834), and among the AD-c patients 2 were autopsied (patients 53 and 400). The neuropathological diagnosis for the 4 AD-hallu patients was AD and DLB for 3 and AD, DLB and medial temporal lobe pathology TDP 43 proteinopathy for 1. The neuropathological diagnosis for the 2 AD-c patients was AD for one and AD, DLB and medial temporal lobe pathology TDP 43 proteinopathy for 1.

Comparison of brain MRI of the 4 AD-hallu patients with AD and DLB to the controls (*P* <0.001, uncorrected, minimum cluster size of 25) showed atrophy of right parahippocampal (BA35), superior temporal gyrus (BA38), left insula, right and left hippocampi, right and left thalami, right anterior cingulate (BA32), and left supramarginal gyrus of the parietal lobe (BA3).

Comparison of brain PET of the 2 AD-hallu patients with AD and DLB to the controls shows hypometabolism (*P*<0.001, uncorrected, minimum cluster size of 25) of right inferior and posterior temporal gyrus (BA 37 and 21), right cuneus of occipital lobe, right precuneus of parietal lobe, right and left gyri of parietal lobes, right and left middle frontal gyri.

## Discussion

The aim of our study was to examine the possible structural and functional neural correlates of hallucinations by contrasting AD hallucinators (AD-hallu) and AD non hallucinators (AD-c). Even though AD-hallu and AD-c were matched for cognitive and functional levels (MMSE, CDR and FAQ), AD-hallu had more extensive brain atrophy and more extensive brain hypometabolism than AD-c. Thus, a wide neuronal network seems to be implicated in the occurrence of hallucinations in AD. We found three groups of regions. The first consisted of “core regions”, with both structural and functional changes, including the right anterior part of the insula and the inferior frontal gyrus next to. The second consisted of regions that were associated with structural changes only: left superior frontal gyrus and lingual gyri, and for intensity, right precentral gyrus, right superior temporal gyrus, and left precuneus. The third consisted of regions with hypoactivity only, comprising a large right ventral and dorsolateral prefrontal area including orbito-frontal regions, and the left midcingulate gyrus for intensity.

The core region underlying hallucinations in patients corresponds to the anterior part of the right insula. The dysfunction of this region could involve different mechanisms leading to hallucinations: (i) false attribution of a source to a stimulus, (ii) a deficient intuitive judgment and (iii) a disruptive processing across attentional networks .

(i) The insular cortex has many connections to the cortex and limbic system. The insula integrates sensory input from the external world and from the internal milieu [31]. The anterior insula – the agranular part of the insula – is known to be extensively connected to limbic areas, higher order visual areas (including lingual gyrus), and olfactory areas [31], [32]. The anterior insular cortex is of high importance for self-awareness and the evaluation of emotional stimuli from others [33], [34]. Thus, dysfunction of the anterior insula would disrupt these two processes, particularly the discrimination between self-generated and external information, and could therefore contribute to hallucinations, as has been suggested in schizophrenia [32]. Goetz et al., have recently suggested that the dysfunction of insula could lead to self-referential visual perceptions, and thus hallucinations [34], [35]

(ii) The anterior insular has certain specific neurons, namely the Von Economo neurons (VENs). These VENs are located in layer 5 of the cingulate gyrus and anterior insular cortex, with a predominance in the right hemisphere [36]. These regions are supposed to be involved in the fast intuitive assessment of complex situations [36]. In situations with a high degree of uncertainty, we do not always have sufficient time to perform deliberative cost-benefit analyses; we need rapid intuitive judgments. In such situations, the cingulate gyrus and anterior insula are active. Allman et al. have suggested that VENs – because of their larger size compared to pyramid neurons – have rapidly conducting axons that may relay a fast intuitive assessment of complex social situations, and so facilitate rapid execution of decisions [36]. Dysfunction of regions of VENs (right predominant anterior insula) – core regions of hallucinations here –could be responsible for non intuitive assessment of external information, including visual information, and could facilitate hallucinations.

(iii) The anterior part of the insula is also involved in high-level cognitive control and attentional processes. The anterior insula is a key region of the "salience network" that permit to segregate the most relevant among internal and external stimuli in order to guide behavior [37]. Anterior insula would permit to switch between networks to facilitate access to attention and working memory resources when a salient event is detected [37]. Thus in PD, patients with PD and high misperceptions and more hallucinations displayed decreased resting state functional connectivity between hubs of the ventral and dorsal attention networks [38].

Among the anterior regions of the network identified in our study, the third group of regions included a large right ventral prefrontal area including the orbito-frontal region, as well as the anterior insula (Figure 3). Along with the insula, the orbito-frontal cortex is known to be of importance for emotion, particularly for negative emotion processing [39]. DLB patients and PD patients with hallucinations have greater GM loss in the right inferior part of the frontal lobe [11]. Moreover, this region was previously found to be correlated to delusional states in AD, particularly the right inferior frontal gyrus [40].

The posterior part of the network of hallucinations in AD includes the lingual gyri and left precuneus. Interestingly, these posterior regions were found after the analysis of the WM but not the GM. The involvement of WM in the posterior part of the brain could be the reflect of a local or more global deconnection of the visual network. The lingual gyri are known to be of importance to process colors, object forms, and complex visual scene [41]. Moreover, they are of importance for the perceptual quality of self-luminosity or glow. At night or during poor lightning, all visible objects appear to glow. And in patients with dementia, low illumination is responsible for a higher rate of visual hallucinations [42]. Such dysfunction of lingual gyri is probably a key point for visual hallucinations or illusions.

Of importance concerning structural changes, we did not find any higher focal brain volume in AD-hallu compared to AD-c, showing that hallucinations are only correlated to atrophy. However, we also found hypermetabolisms with a predominant left side of the brain pattern. We suppose that these predominant left hypermetabolisms are compensatory phenomena, including the left superior frontal gyrus, the left fusiform, and postcentral gyri.

Only one previous study evaluated 16 visual hallucinations of a patient with a neurodegenerative disease (Parkinson's disease) during fMRI scan. This subject showed significantly less cortical activation in the occipital visual cortex (right lingual gyrus) and markedly increased activation in the bilateral cingulate and insula, right medial frontal gyrus, right postcentral gyrus, right thalamus and brainstem [35]. These regions are coherent with those found in our study.

We have observed differences between the functional PET analysis and the structural MRI analysis. These differences could be due to the fact that the patients with the FDG-PET represent the half of the whole AD-hallu group. But more probably, these differences are due to the fact that usually in AD the hypometabolism is more important than the atrophy [43] Another reason could be du to the fact that a part of the hallucinated patients have also a DLB (see infra): DLB is reponsible for synaptic dysfunction (functional) more than neuron destruction (structural) [44].

The results of this study are coherent with the top-down/bottom-up hypotheses on hallucinations [16] since we have found key region for attention (i.e. the anterior part of the insula), and also a large part of the right frontal lobe with respectively structural and functional, and functional involvement, compatible with impaired attentional binding, and atrophy of the lingual gyri compatible with perceptual impairment. The results of this study are also compatible with the hypotheses of Shine et al., since the involvement of the anterior insula as a core region of hallucinations could be responsible for a conflict resolution processed by neural network unprepared to do it [19]: default mode network (DMN) and ventral attention network (VAN) also named salience network. Moreover, recently Shine et al., have demonstrated that PD patients with hallucinations compared to PD patients without have the same atrophy found here i.e. clear atrophy of the anterior part of the insula [38]. The anterior insula is proposed to act in conjunction with cortical and sub-cortcal hubs within the VAN to activate the Dorsal Anterior Network (DAN) in the presence of environmental salience. Patients with atrophy in the anterior insula may have lost the ability to effectively “switch” [45] their attention in the presence of an ambiguous stimulus, leading to an inability to appropriately activate the DAN [38].

The question of the diagnosis in all studies on cognitive neurodegenerative diseases is of importance. Here we used the research Dubois' criteria for the diagnosis of the patients [23]. These criteria have a known specificity of 93% [22], that is a very good specificity. Moreover, 62% of the AD patients with hallucinations have had a CSF analysis and all these patients have CSF results in favor of AD. However, some of these patients have probably AD and the diagnosis of DLB. Diagnostic classification of DLB is based on McKeith's criteria with core diagnostic features of DLB being (1) recurrent visual hallucinations, (2) cognitive fluctuations, and (3) spontaneous motor features of parkinsonism [46]. The presence of one of these core signs is sufficient for a diagnosis of possible DLB, and two for probable DLB [46]. Interestingly the McKeith's criteria have an excellent specificity (more than 95%) [47], [48] when compared to gold standard neuropathological diagnosis. Against the AD and DLB hypothesis, we have the poor percentage of parkinsonism in our cohort, but we don't have any information in ADNI about fluctuations [46], and the presence of hallucinations and dementia in our AD-hallu group is sufficient to diagnose a possible DLB. Previous neuropathological analysis in a subset of the ADNI cohort showed that among 22 patients with the diagnosis of AD (MCI or dementia), only seven had a pure AD [30]. The majority of the patients had mixed pathology including AD and LBD (n = 6), AD and medial temporal lobe pathology (MTLP: TDP-43 proteinopathy, argyrophilic grain disease and hippocampal sclerosis) (n = 5), and AD and DLB and MTLP (n = 4). Moreover in this study, hallucinations were a strong predictor of coincident DLB (specificity = 100%). We have checked the neuropathological status of the patients of our study using the article of Toledo et al., [30] and we have found that the 4 patients autopsied in the AD-hallu group have the mixed pathology AD and DLB. So it is highly probable that hallucinations studied here are secondary not only to AD but AD and DLB, and that the neural basis of hallucinations here are those of AD and DLB. Interestingly, anterior part of the insula was recently recognized as a biomarker of DLB. Indeed, a metanalysis of gray matter atrophy in DLB using VBM was conducted [49]. Seven studies including 218 DLB patients compared to 219 controls showed an insular cortex atrophy predominantly on the right in DLB patients.

The first limitation of our study is the absence of details on hallucinations because only the NPI-Q was used. Two types of information were not known: the type and the occurrence of hallucinations. Thus, it is not known whether the subjects were actively hallucinating during the FDG-PET scans. It is worth noting that there may be differences in anatomical and functionnal patterns identified in imaging studies associated with chronic hallucinations versus the rare occurrence of capturing acute hallucinations [35]. However, even if there are no clinical data in the ADNI on the type of hallucinations in patients, it is known that in AD visual hallucinations are more frequent than auditory verbal hallucinations [16]. Moreover, since hallucinations are less frequent in AD than in other neurodegenerative diseases, such as DLB, the only way that one can study so many patients with AD and hallucinations is to have recourse to a cohort as large as the ADNI. The second limitation is that, among the patients with brain MRI (n = 39), only a half had brain FDG-PET. Thus, the "core region" reflect a much smaller subset of patients, and this could introduce substantial bias. This limitation is inherent in the ADNI cohort, in which only about half of the patients have FDG-PET.

In our study, the presence of hallucinations in AD (or AD and DLB) was correlated with a vast anterior-posterior network, with a predominant right brain involvement. We suppose that patients have a top-down control failure, and also a dysfunction of the bottom-up visual processes. Top-down mechanisms are underlain by the right anterior insula and the right frontal lobe known to be involved in false attribution of a source to a stimulus and deficient intuitive judgment. Bottom-up mechanisms are involved in visuo-perceptual processing with posterior regions. Taken together, all these data are consistent with previously published data on hallucinations in neurodegenerative diseases. Further studies are now needed to elucidate more precisely the anatomic and functional connections underlying hallucinations in AD patients.
